# Pathogen profiles and risk factors of hospital-acquired infections in traumatic brain injury patients

**DOI:** 10.3389/fpubh.2026.1723961

**Published:** 2026-01-15

**Authors:** Tingting Liu, Huan Zhao, Hongyu Huo, Huiting Zhong, Ruiling Wen

**Affiliations:** 1 Department of Infection Management, Huizhou First Hospital, Huizhou, Guangdong, China; 2 Nursing, Health College, Guangzhou Huashang University, Guangzhou, Guangdong, China

**Keywords:** hospital-acquired infections, infection control, pathogen profiles, risk factors, traumatic brain injury

## Abstract

**Background:**

Traumatic brain injury (TBI) represents a significant public health challenge, marked by elevated incidence rates and significant morbidity, frequently resulting in long-term disabilities and imposing substantial economic burdens on healthcare systems. This study seeks to examine the incidence of hospital-acquired infections (HAIs), characterize prevalent pathogens, evaluate antimicrobial susceptibility patterns, and identify risk factors associated with HAIs in TBI patients.

**Methods:**

A retrospective analysis was performed on a cohort of 2,314 hospitalized TBI patients. Data were collected on demographic characteristics, clinical history, treatment modalities, and infection outcomes were systematically collected. The incidence proportion of HAIs in TBI patients was analyzed, key pathogens and their antimicrobial resistance patterns were identified. Univariate and multivariate logistic regression analyses were performed to assess risk factors for HAIs of TBI patients.

**Results:**

Among 2,314 hospitalized TBI patients, the incidence of HAIs was 19.40%. The most frequent infection sites were the lower respiratory tract (14.87%) and urinary tract (6.66%), whereas gentral nervous system infections were infrequent (0.39%). A total of 555 distinct pathogens were first-isolated, with Gram-negative bacteria representing the majority (66.85%), particularly *Klebsiella pneumoniae* (14.59%) and *Pseudomonas aeruginosa* (11.17%). Significant antimicrobial resistance was observed, including 100% resistance of *Klebsiella pneumoniae* to ampicillin and 95.83% resistance of *Acinetobacter baumannii* to ceftazidime. Univariate and multivariate logistic regression analyses revealed several independent risk factors for HAIs in TBI patients, including age ≥60 years, Glasgow Coma Scale (GCS) score ≤8, hospitalization duration exceeding 2 weeks, and the presence of urinary catheters.

**Conclusion:**

This study highlights the necessity of risk factor identification for HAIs to facilitate risk stratification and the implementation of targeted preventive measures in the clinical management of TBI patients. Furthermore, sustained microbial surveillance is imperative to guide antibiotic stewardship and enhance diagnostic and therapeutic strategies for this patient population.

## Introduction

Traumatic brain injury (TBI) represents a critical public health issue, characterized by a high incidence rate and significant morbidity. The consequences of TBI extend beyond immediate physical injuries, often leading to long-term disabilities and substantial economic burdens on healthcare systems (1, 2). TBI is a complex condition that can result from various causes, including falls, vehicle accidents, and sports-related injuries, and its management poses substantial challenges for clinicians (3). Despite advancements in diagnostic and therapeutic approaches, including various imaging techniques and surgical interventions, challenges remain. Key limitations include delayed diagnosis, inadequate infection control measures, and the potential for hospital-acquired infections (HAIs) that can complicate patient recovery (4, 5). Therefore, there is a pressing need to explore the complexities of TBI and its associated complications, particularly HAIs.

Recent studies have highlighted the prevalence of HAIs as a common complication among hospitalized patients, significantly affecting their recovery trajectories and mortality rates (6). This underscores the critical intersection between TBI and HAIs, emphasizing the necessity for effective infection prevention strategies tailored for this vulnerable population. Understanding the interplay between TBI and HAIs can inform clinical practices and enhance patient outcomes, further reflecting the importance of this research avenue (7–9). Previous studies on the pathogen spectrum, drug resistance characteristics and related risk factors of infections in TBI patients are still relatively limited. There is an urgent need for more in-depth exploration and analysis to provide more effective guidance for clinical treatment.

This study employs a retrospective analysis methodology, utilizing existing patient data to identify trends and outcomes associated with HAIs in TBI patients. This approach allows for the examination of a considerable dataset derived from real clinical scenarios, providing a robust foundation for analysis. By focusing on evaluating the incidence of HAIs in TBI patients, identifying the most common pathogens involved, assessing their antimicrobial susceptibility, and exploring independent risk factors that contribute to the development of these infections. The insights gained from this study are anticipated to inform clinical practices, enhance preventive measures, and ultimately improve the patient outcomes in this vulnerable population. Understanding the specific risk factors associated with HAIs in TBI patients is crucial for the development of targeted strategies that can mitigate these risks and improve the quality of care provided to these individuals.

## Materials and methods

### Study setting

A retrospective analysis was conducted, selecting patients with traumatic brain injury (TBI) who were hospitalized at Huizhou First Hospital from January 2018 to December 2024 as the study subjects. Inclusion criteria: All patients had acute head trauma, and their cranial CT examinations met the diagnostic criteria for traumatic brain injury, with a hospitalization duration exceeding 48 h. Exclusion criteria: (1) Patients with a history of neurological diseases or prior head trauma. (2) Patients who were already infected or in the incubation period of infection before or at the time of admission. (3) Patients who had been treated at other hospitals for trauma and had undergone surgery.

### Data collection

Clinical data were collected through the real-time automatic nosocomial infection surveillance system (Xinglin Technology, Hangzhou, China), including: (1) Sociodemographic data such as sex, age, and medical history (hypertension, diabetes). (2) Disease-related data, including Glasgow Coma Scale (GCS) and whether the traumatic brain injury was complicated by chest, abdominal, limb, spinal, or pelvic injuries. (3) Treatment data, including surgical treatment, ventilation use, central venous catheter insertion, urinary catheterization, and antibiotic usage.

### Definitions

Hospital-acquired infections (HAIs) were defined according to the *Nosocomial Infection Diagnostic Criteria* in 2001 published by Ministry of Public Health of People’s Republic of China (10). Hospital-acquired infections (HAIs) were defined as infections that were not present or incubating at the time of hospital admission, but manifested 48 h or more after admission. Infections identified at admission or within the first 48 h were considered community-acquired and excluded. HAIs were classified based on the infection site, including respiratory infections, urinary tract infections, gastrointestinal infections, surgical site infections, bloodstream infections, abdominal infections, meningitis, skin and soft tissue infections, and other infections. Multidrug-resistant organisms (MDROs) refer to bacteria that demonstrate simultaneous resistance to three or more categories of antimicrobial agents employed in clinical settings. And this study included the key monitored MDROs, including carbapenem-resistant Enterobacteriaceae (CRE), methicillin-resistant *Staphylococcus aureus* (MRSA), vancomycin-resistant Enterococcus (VRE), carbapenem-resistant *Acinetobacter baumannii* (CRAB), and carbapenem-resistant *Pseudomonas aeruginosa* (CRPA).

### Microbiological testing

Collection, submission, and culture of clinical specimens (such as sputum, blood, urine, cerebrospinal fluid, etc.) followed standard operating procedures. Specimens were taken for examination, and microbial culture and drug sensitivity tests were conducted. Antimicrobial susceptibility testing (AST) was performed using the disk diffusion method or by determining the minimum inhibitory concentration (MIC). All AST procedures and interpretations were performed in strict accordance with the Clinical and Laboratory Standards Institute (CLSI) guidelines M100, 28th–33rd version (11) from 2018 to 2024. To avoid duplicate entries, only the first isolate of a given bacterial species per patient was included in the final analysis, regardless of the antibiogram. For quality control, reference strains including *Escherichia coli* (ATCC 25922), *Staphylococcus aureus* (ATCC 25923), *Pseudomonas aeruginosa* (ATCC 27853) and *Staphylococcus faecalis* (ATCC 29212) were used routinely.

### Data analysis

Data analysis was performed using IBM SPSS Statistics version 22.0 software (IBM Corp., Armonk, N. Y., USA). Count data were expressed as frequency (n) and prevalence proportion (%). The distribution of pathogens and susceptibility results was primarily analyzed using descriptive statistical methods. To investigate potential risk factors for HAIs in TBI patients, univariate analysis was first conducted: categorical variables between the HAI group and non-HAI group were compared using the χ^2^ test. Variables with *p* < 0.05 in univariate analysis and those with clinical significance were included in multivariate logistic regression analysis, using forward likelihood ratio method for variable selection, with the occurrence of HAI as the dependent variable (Yes = 1, No = 0). Variance inflation factors (VIF) were calculated for all independent variables included in the multivariate logistic regression model. VIF < 5 indicated the absence of severe collinearity. Two-tailed *p* values <0.05 were considered statistically significant and had a 95% confidence interval (CI). The goodness-of-fit test was used to assess the multivariate logistic regression model. *p*-values greater than 0.05 for the Pearson chi-square test and the deviance chi-square test indicated adequate model fit.

## Results

### The rates of HAIs and site-specific HAIs of TBI patients

A total of 2,314 patients with traumatic brain injury were included in this study, with 449 cases of hospital-acquired infections, resulting in an infection rate of 19.40%. The primary infection site was the lower respiratory tract, with a total of 344 cases (14.87%), including 93 cases of ventilator-associated pneumonia (VAP) (4.02%). There were 154 cases of urinary tract infections (6.66%), half of which were catheter-associated infections. Other common infection sites included skin and soft tissue (34 cases, 1.47%), bacteremia (43 cases, 1.86%), and catheter-related bloodstream infections (20 cases, 0.86%). There were a total of 9 cases (0.39%) of gentral nervous system infections. The incidence of infections at other sites was below 0.5% (Table 1). Regarding device utilization, the ventilator use rate was 17.68% (409/2314), with a total of 4,611 ventilator-days and a median duration of 9 days; the VAP rate was 20.02 per 1,000 ventilator-days. The central venous catheter (CVC) use rate was 30.73% (711/2314), with 14,014 catheter-days and a median duration of 13 days; the catheter-related bloodstream infection rate was 1.43 per 1,000 catheter-days. The urinary catheter use rate was 54.41% (1,259/2314), with 22,652 catheter-days and a median duration of 11 days; the catheter-associated urinary tract infection (CAUTI) rate was 20.02 per 1,000 catheter-days.

**Table 1:** The prevalence proportion of HAIs and site-specific HAIs of TBI patients.

HAIs site	Infection cases	Prevalence proportion %
HAIs	449	19.40
Lower respiratory tract infections	344	14.87
Non-VAP	251	10.85
VAP	93	4.02
Urinary system infections	154	6.66
Non-CAUTI	77	3.33
CAUTI	77	3.33
Blood stream infections	43	1.86
Non-CLABSI	23	0.99
CLABSI	20	0.86
Skin and soft tissue infections	34	1.47
Upper respiratory tract infections	11	0.48
Gentral nervous system infections	9	0.39
Surgical site infections	7	0.30
Abdominal infection	4	0.17
Gastroenteritis	2	0.09
Pleurisy	2	0.09
Osteomyelitis	1	0.04
Oral infection	1	0.04
Other infections	5	0.22

HAI, hospital-acquired infection; TBI, traumatic brain injury; CAUTI, catheter associated urinary tract infections; CLABSI, central line associated bloodstream infections; VAP, ventilator associated pneumonia.

### Pathogen distribution of patients with HAI

During the study period, a total of 555 unique pathogens were first-isolated from infection specimens of TBI patients who met the criteria. Gram-negative bacteria were the predominant pathogens, with 371 strains, accounting for 66.85% of the total. This was followed by fungi (94 strains, 16.94%) and Gram-positive bacteria (90 strains, 16.22%). Among Gram-negative bacteria, the top four detected pathogens were *Klebsiella pneumoniae* (81 strains, 14.59%), *Pseudomonas aeruginosa* (62 strains, 11.17%), *Acinetobacter baumannii* (48 strains, 8.65%), and *Escherichia coli* (44 strains, 7.93%). Among Gram-positive bacteria, *Staphylococcus aureus* (47 strains, 8.47%) and *Enterococcus faecalis* (19 strains, 3.42%) were predominant. The most common fungi were *Candida albicans* (33 strains, 5.95%) and *Candida glabrata* (32 strains, 5.77%) (Table 2). Among them, the detection rates of CRE, MRSA, VRE, CRAB, and CRPA in the key monitored multi-drug resistant bacteria were 4.84% (5 strains), 57.45% (27 stains), 4.00% (2 stains), 20.83% (10 strains), and 24.19% (15 strains).

**Table 2:** Organisms identified from patients with HAI.

Organism	Number of strains	Percentage %
Gram negative	**371**	**66.85**
*Klebsiella pneumoniae*	81	14.59
*Pseudomonas aeruginosa*	62	11.17
*Acinetobacter baumannii*	48	8.65
*Escherichia coli*	44	7.93
*Stenotrophomonas maltophilia*	35	6.31
*Enterobacter aerogenes*	19	3.42
*Proteus mirabilis*	12	2.16
*Enterobacter cloacae*	12	2.16
*Burkholderia asburiae*	9	1.62
*Haemophilus influenzae*	8	1.44
*Serratia marcescens*	8	1.44
*Citrobacter koseri*	6	1.08
*Klebsiella acidophilus*	5	0.90
*Moraxella catarrhalis*	3	0.54
Other gram negative organisms	19	3.42
Gram positive	**90**	**16.22**
*Staphylococcus aureus*	47	8.47
*Enterococcus faecium*	19	3.42
*Staphylococcus epidermidis*	6	1.08
*Enterococcus faecalis*	6	1.08
*Staphylococcus hemolyticus*	5	0.90
*Staphylococcus capitis*	3	0.54
Other gram positive organisms	4	0.72
Fungi	**94**	**16.94**
*Candida albicans*	33	5.95
*Candida glabrata*	32	5.77
*Candida tropicalis*	21	3.78
Other fungi	8	1.44
Total	**555**	**100**

Bold values indicate the total number and percentage for major organism categories (Gram negatives, Gram positives, Fungi).

### Antibiotic resistance rate of the major species of Gram-negative bacteria

Antibiotic susceptibility testing showed that *Klebsiella pneumoniae* was highly sensitive to meropenem (97.53%) and imipenem (95.06%), with a sensitivity rate of 91.36% to piperacillin/tazobactam. However, it had a resistance rate of 100% to ampicillin. *Escherichia coli* also maintained high sensitivity to carbapenems (imipenem and meropenem) (>95%), but had high resistance rates to ampicillin (88.37%) and tetracycline (79.07%). *Acinetobacter baumannii* exhibited severe resistance issues, with a resistance rate of 95.83% to ceftazidime and nearly 30.00% to carbapenems. It was relatively sensitive to levofloxacin (83.33%) and colistin (75.00%). *Pseudomonas aeruginosa* was most sensitive to colistin (100%) and amikacin (96.77%), but showed 100% resistance to tetracycline, ampicillin, trinethoprin/sulfameth, piperacillin, chloramphenicol and ceftazidime (Table 3).

**Table 3:** Antibiotic resistance rate of the major species of Gram-negative bacteria.

Antimicrobial agent	*Klebsiella pneumoniae* (*n* = 81)		*Escherichia coli* (*n* = 43)		*Acinetobacter baumannii* (*n* = 48)		*Pseudomonas aeruginosa* (*n* = 62)	
	No. of Drug-resistant strains	Drug resistance rate (%)	No. of Drug-resistant strains	Drug resistance rate (%)	No. of Drug-resistant strains	Drug resistance rate (%)	No. of Drug-resistant strains	Drug resistance rate (%)
Piperacillin/Tazobactam	7	8.64	3	6.98	10	20.83	3	4.84
Tetracycline	25	30.86	34	79.07	22	45.83	62	100.00
Levofloxacin	7	8.64	21	48.84	8	16.67	7	11.29
Moxifloxacin	8	9.88	21	48.84	1	2.08	/	/
Aztreonam	14	17.28	20	46.51	48	100.00	11	17.74
Imipenem	4	4.94	1	2.33	10	20.83	15	24.19
Meropenem	2	2.47	2	4.65	14	29.17	10	16.13
Ampicillin	81	100.00	38	88.37	48	100.00	62	100.00
Trinethoprin/Sulfameth	20	24.69	18	41.86	9	18.75	62	100.00
Ampicillin/Sulbact	21	25.93	36	83.72	13	27.08	4	6.45
Piperacillin	15	18.52	19	44.19	44	91.67	62	100.00
Chloramphenicol	25	30.86	30	69.77	48	100.00	62	100.00
Cefazolin	3	3.70	0	0.00	11	22.92	3	4.84
Amikacin	14	17.28	25	58.14	10	20.83	2	3.23
Ciprofloxacin	15	18.52	14	32.56	10	20.83	8	12.90
Ceftazidime	12	14.81	10	23.26	46	95.83	62	100.00
Amoxicillin/Clavulanic acid	19	23.46	22	51.16	11	22.92	6	9.68
Cefepime	0	0.00	0	0.00	1	2.08	0	0.00
Colistin	9	11.11	16	37.21	12	25.00	0	0.00
Gentamicin	20	24.69	26	60.47	10	20.83	62	100.00
Cefotaxime	7	8.64	3	6.98	10	20.83	3	4.84

### Antimicrobial sensitivity of major Gram-positive bacteria

Among *Staphylococcus aureus*, the resistance rate to cefoxitin (i.e., MRSA detection rate) was 57.45%. No strains resistant to gentamicin, trimethoprim, erythromycin, fusidic Acid, linezolid or nitrofurantoin were found (sensitivity rate 100%). However, it showed high resistance rates to tobramycin (95.74%), rifampin (95.74%), teicoplanin (89.36%), ampicillin and amoxicillin/clavulanic acid (53.19%). Coagulase-negative staphylococci had an even higher resistance rate to tobramycin (100%), ampicillin (81.82%) and amoxicillin/clavulanic acid (63.64%), but were 100% sensitive to gentamicin, trimethoprim, fusidic Acid, and nitrofurantoin (Table 4).

**Table 4:** Antibiotic resistance of the major species of gram-positive bacteria.

Antimicrobial agent	*Staphylococcus aureus* (*n* = 47)		Coagulase-negative staphylococcus (*n* = 11)	
	No. of Drug-resistant strains	Drug resistance rate (%)	No. of Drug-resistant strains	Drug resistance rate (%)
Gentamicin	0	0.00	0	0.00
Amikacin	31	65.96	11	100.00
Tobramycin	45	95.74	11	100.00
Cefoxitin	27	57.45	10	90.91
Ampicillin	25	53.19	9	81.82
Penicillin	9	19.15	7	63.64
Amoxicillin/Clavulanic acid	25	53.19	7	63.64
Trimethoprim/Sulfameth	2	4.26	2	18.18
Trimethoprim	0	0.00	0	0.00
Teicoplanin	42	89.36	11	100.00
Vancomycin	5	10.64	6	54.55
Clindamycin	12	25.53	3	27.27
Erythromycin	0	0.00	0	0.00
Quinupristin/Dalfopristin Syncercid	2	4.26	2	18.18
Fusidic Acid	0	0.00	0	0.00
Linezolid	0	0.00	2	18.18
Nitrofurantoin	0	0.00	0	0.00
Ciprofloxacin	31	65.96	11	100.00
Rifampin	45	95.74	11	100.00
Tetracycline	27	57.45	10	90.91

### Univariate analysis on potential risk factors for HAI of TBI patients

Univariate analysis results showed (Table 5) that there were statistically significant differences in the distribution of multiple variables between the HAI group (*n* = 449) and the non-HAI group (*n* = 1865) (*p* < 0.05). Factors significantly associated with HAI included: age ≥60 years (35.63% vs. 20.32%, *p* < 0.001), GCS score ≤8 (57.02% vs. 5.74%, *p* < 0.001), hospitalization over 2 weeks (85.08% vs. 34.26%, *p* < 0.001), surgical intervention (67.93% vs. 34.48%, *p* < 0.001), surgical operation lasted over 3 h (30.73% vs. 11.05%, *p* < 0.001), having suffered more than two surgeries (34.08% vs. 8.74%, *p* < 0.001), prescription of antibiotic (96.88% vs. 68.58%, *p* < 0.001), antibiotic used over 2 weeks (78.62% vs. 19.95%, *p* < 0.001), antimicrobial combination (87.31% vs. 32.71%, *p* < 0.001), as well as catheter placement (tracheal intubation/tracheostomy, urinary catheter, CVC) at admission and over 2 weeks (all *p* < 0.001). Regarding comorbidities, pulmonary emphysema (5.35% vs. 3.32%, *p* = 0.042), renal insufficiency (2.90% vs. 0.86%, *p* < 0.001), hypohepatia (3.79% vs. 1.18%, *p* < 0.001), hypertension (12.47% vs. 9.06%, *p* = 0.029), and diabetes mellitus (6.90% vs. 4.24%, *p* = 0.017) showed significant differences between the two groups. Additionally, multiple trauma involving the chest, abdomen, spine, pelvis, etc., was also associated with HAI occurrence (all *p* < 0.05). However, male (*p* = 0.418) and coronary heart disease (*p* = 0.617) showed no significant differences between the two groups (Table 5).

**Table 5:** Univariate analysis on potential risk factors for HAI of TBI patients.

Comparison	HAI (*n* = 449)	Non-HAI (*n* = 1865)	χ^2^	*p*
Age ≥60 years (*n*/%)	160 (35.63)	379 (20.32)	47.492	<0.001
Male (*n*/%)	334 (74.39)	1,352 (72.49)	0.657	0.418
GCS ≤ 8 (*n*/%)	256 (57.02)	107 (5.74)	719.564	<0.001
Hospitalization over 2 weeks (*n*/%)	382 (85.08)	639 (34.26)	379.011	<0.001
Surgical intervention (*n*/%)	305 (67.93)	643 (34.48)	167.441	<0.001
Surgical operation lasted over 3 h (*n*/%)	138 (30.73)	206 (11.05)	110.848	<0.001
Having suffered more than two surgeries (*n*/%)	153 (34.08)	163 (8.74)	197.004	<0.001
Prescription of antibiotic (*n*/%)	435 (96.88)	1,279 (68.58)	150.934	<0.001
Antibiotic used exceeding two weeks (*n*/%)	353 (78.62)	372 (19.95)	579.028	<0.001
Antimicrobial combination (*n*/%)	392 (87.31)	610 (32.71)	439.370	<0.001
Catheter at admission (*n*/%)				
Tracheal intubation/tracheostomy at admission	238 (53.01)	171 (9.17)	477.933	<0.001
Tracheal intubation/tracheostomy at admission and over 2 weeks	71 (15.81)	22 (1.18)	200.882	<0.001
Urinary catheter at admission	425 (94.65)	834 (44.72)	363.783	<0.001
Urinary catheter at admission and over 2 weeks	323 (71.94)	234 (12.55)	698.391	<0.001
CVC at admission	312 (69.49)	399 (21.39)	393.243	<0.001
CVC at admission and over 2 weeks	213 (47.44)	127 (6.81)	476.581	<0.001
Comorbidities (*n*/%)				
Pulmonary emphysema	24 (5.35)	62 (3.32)	4.130	0.042
Coronary heart disease	9 (2.00)	31 (1.66)	0.250	0.617
Renal insufficiency	13 (2.90)	16 (0.86)	12.138	<0.001
Hypohepatia	17 (3.79)	22 (1.18)	14.838	<0.001
Hypentersion	56 (12.47)	169 (9.06)	4.795	0.029
Diabetes mellitus	31 (6.90)	79 (4.24)	5.691	0.017
Multi-site trauma (*n*/%)				
With chest	170 (37.86)	548 (29.38)	12.156	<0.001
With abdominal	38 (8.46)	89 (44.77)	9.505	0.002
With extremity	62 (13.81)	196 (10.51)	3.976	0.046
With spine	54 (12.03)	137 (7.35)	10.470	<0.001
With basin	29 (6.46)	79 (4.24)	4.019	0.045

HAI, hospital-acquired infection; TBI, traumatic brain injury; GCS, Glasgow Coma Scale; CVC, central venous catheter.

### Multivariate logistics regression analysis on potential risk factors for HAI of TBI patients

Variables with meaningful significance from univariate analysis were included in the multivariate logistic regression model. The VIF values ranged from 1.013 to 2.955, all well below the commonly used threshold of 5, indicating that multicollinearity was not a substantial issue. The results showed (Table 6) that age ≥60 years (OR = 1.887, 95% CI: 1.322–2.695), GCS score ≤8 (OR = 10.952, 95% CI: 7.535–15.920), hospitalization over 2 weeks (OR = 3.919, 95% CI: 2.633–5.833), having suffered more than two surgeries (OR = 1.686, 95% CI: 1.107–2.568), prescription of antibiotic (OR = 16.940, 95% CI: 2.270–126.424), antibiotic used over 2 weeks (OR = 1.697, 95% CI: 1.072–2.686), antimicrobial combination (OR = 2.419, 95% CI: 1.512–3.872), urinary catheter at admission (OR = 2.823, 95% CI: 1.585–5.027), and urinary catheter at admission and over 2 weeks (OR = 2.022, 95% CI: 1.329–3.078) were independent risk factors for HAI occurrence in TBI patients (all *p* < 0.05). Factors such as surgical treatment, surgical duration, tracheal intubation/tracheostomy at admission and CVC at admission did not show independent correlations in multivariate analysis (*p* > 0.05) (Table 6). Regarding the goodness-of-fit test results, *p* values of the chi-square test of Pearson and the chi-square test of Deviance were 0.082 and 1.000, all greater than 0.05, indicating that the model fit was good.

**Table 6:** Multivariate logistics regression analysis on potential risk factors for HAI of TBI patients.

Comparison	β	χ^2^	OR	95%CI	*p*
Age ≥60 years	0.182	12.202	1.887	1.322–2.695	<0.001
GCS ≤ 8	0.191	157.322	10.952	7.535–15.920	<0.001
Hospitalization over 2 weeks	0.203	45.292	3.919	2.633–5.833	<0.001
Surgical intervention	0.215	2.840	0.696	0.456–1.061	0.092
Surgical operation lasted over 3 h	0.207	1.559	0.772	0.515–1.158	0.212
Having suffered more than two surgeries	0.215	5.927	1.686	1.107–2.568	0.015
Prescription of antibiotic	1.026	7.613	16.940	2.270–126.423	0.006
Antibiotic used over two weeks	0.234	5.089	1.697	1.072–2.686	0.024
Antimicrobial combination	0.240	13.563	2.419	1.512–3.872	<0.001
Tracheal intubation/tracheostomy at admission	0.214	0.805	1.212	0.796–1.845	0.369
Tracheal intubation/tracheostomy at admission and over 2 weeks	0.342	0.199	1.165	0.596–2.277	0.656
Urinary catheter at admission	0.294	12.425	2.823	1.585–5.027	<0.001
Urinary catheter at admission and over 2 weeks	0.214	10.811	2.022	1.329–3.078	0.001
CVC at admission	0.241	1.083	0.778	0.485–1.248	0.298
CVC at admission and over 2 weeks	0.230	1.918	1.376	0.876–2.161	0.166
Pulmonary emphysema	0.381	3.055	1.945	0.922–4.101	0.080
Renal insufficiency	0.630	0.883	0.553	0.161–1.903	0.347
Hypohepatia	0.530	1.816	2.043	0.723–5.775	0.178
Hypentersion	0.268	1.001	1.308	0.773–2.213	0.317
Diabetes mellitus	0.362	0.962	1.426	0.702–2.899	0.327
TBI with chest trauma	0.181	7.993	0.599	0.420–0.855	0.005
TBI with abdominal trauma	0.313	0.106	0.903	0.489–1.668	0.745
TBI with extremity trauma	0.228	0.638	0.833	0.533–1.304	0.425
TBI with spine trauma	0.249	0.323	0.868	0.533–1.413	0.570
TBI with basin trauma	0.328	1.649	0.656	0.345–1.248	0.199

HAI, hospital-acquired infection; TBI, traumatic brain injury; GCS, Glasgow Coma Scale; CVC, central venous catheter.

## Discussion

TBI is a serious medical condition arising from external factors that induce damage to brain tissue, commonly occurring in situations such as traffic accidents, falls, and sports injuries (3). This condition exerts substantial effects on patients’ physical health and may be associated with a range of psychological and social challenges, imposing to considerable economic burdens for patients and their families (2, 3). With the growing prevalence of transportation and social activities, the incidence of TBI has demonstrated an increasing trend, accompanied by persistently elevated mortality and disability rates (2, 3, 12). Consequently, comprehensive investigations into the pathogen spectrum, antibiotic resistance profiles, and associated risk factors for infections in TBI patients are critically needed to inform clinical management and enhance patient outcomes.

This study aims to systematically analyze the incidence proportion of hospital-acquired infections in hospitalized TBI patients, identify the predominant pathogenic bacteria and their resistance characteristics, and thereby establish a scientific foundation for infection prevention and treatment. Through a retrospective analysis of large-sample data, the primary pathogenic bacteria responsible for infections were identified, and correlated risk factors were explored. These findings provide quantitative evidence to support clinical decision-making, fostering improved understanding of infection management in TBI patients and the formulation of targeted interventions.

In this study, we observed a significant association between the age of TBI patients and infection risk, specifically reflected in a higher infection rate among older individual patients. This observation aligns with prior research, as multiple studies have reported that older population, in the context of age-related immune decline, often demonstrate heightened susceptibility to infections (12, 13). At the molecular level, advancing age is accompanied by a gradual weakening of immune response capacity, particularly in cellular immune function, which may reduce resistance to bacterial infections (12, 13). Additionally, older population frequently present with multiple comorbidities, such as diabetes and hypertension, which can further exacerbate infection risk (14). In contrast, younger patients typically exhibit more robust immune systems, potentially allowing more effective responses to infections. Thus, infection prevention measures for older population warrant heightened stringency to mitigate infection rates.

Regarding pathogenic bacteria, relevant studies indicate that Gram-negative bacteria predominate in hospital-acquired infections following TBI, with *Pseudomonas aeruginosa* and Klebsiella species commonly identified in severe TBI cases (15, 16). Our results are consistent with these reports, as detection rates for *Klebsiella pneumoniae* and *Pseudomonas aeruginosa* were 14.59 and 11.17%, respectively. Antibiotic resistance in these bacteria has shown an increasing trend, complicating therapeutic approaches. In this study, CRPA constituted 24.19% of detected *Pseudomonas aeruginosa* isolates. Furthermore, the notable detection rate of *Staphylococcus aureus* merits attention. Previous studies have indicated that *Staphylococcus aureus* can exhibit high detection rates in certain settings (15, 17), suggesting that in TBI patients, particularly those undergoing surgical procedures, vigilance regarding this pathogen is warranted. In this cohort, *Staphylococcus aureus* was detected in 8.47% of TBI patients, with MRSA accounting for 57.45% of *Staphylococcus aureus* isolates and 4.86% of the total 555 detected strains. Literature suggests that MRSA prevalence is closely tied to antibiotic utilization and selective pressures within hospital environments (18, 19). This resistance pattern not only complicates treatment regimens but may also correlate with higher rates of therapeutic failure. Studies (18, 19) note that MRSA infections are associated with factors such as prolonged hospitalization, surgical history, and antibiotic exposure, which resonate with the risk factors identified in our analysis. This underscores the need for comprehensive evaluation of antibiotic use strategies in hospital infection control to curb the dissemination of resistant bacteria.

In terms of antibiotic resistance, this study documented the key pathogens and their antimicrobial resistance patterns mirror global trend of increasing antibiotic resistance, with numerous studies linking resistance increases to antibiotic overuse and inappropriate application (20). Mechanistically, bacteria develop resistance through genetic mutations, horizontal gene transfer, and other processes (21). The complexity of these mechanisms presents significant challenges to antibiotic efficacy (21). Particularly in critically ill patients, suboptimal infection control measures and imprudent antibiotic use may exacerbate the selection and spread of resistant strains (22, 23). Therefore, the implementation of rational antibiotic stewardship strategies is of paramount importance.

In assessing hospital-acquired infection risk in TBI patients, injury severity represents a critical factor. Our analysis indicated that lower GCS scores are associated with elevated infection risk. Previous research corroborates that patients with lower GCS scores exhibit significantly increased infection probabilities during hospitalization, which may relate to impaired neurological function and compromised immune responses (24–26). Our findings emphasize the role of comorbidities in infection risk, particularly the immune-suppressive effects of conditions such as diabetes and hypertension, consistent with prior literature (12, 27, 28). Diabetic patients, often experiencing immune dysfunction due to poor glycemic control, may display increased infection susceptibility (29, 30). Patients with extended hospital stays also face heightened infection risk, potentially attributable to nosocomial transmission of resistant bacteria and exposure to invasive procedures (12, 31). Thus, early intervention and monitoring for patients with comorbidities are essential components of infection risk reduction.

The findings of this study underscore critical areas for intervention to reduce HAIs in TBI patients. Based on identified risk factors such as advanced age, comorbidities, prolonged hospitalization, and antimicrobial resistance patterns, we propose the following measures for hospitals and healthcare providers. Enhanced surveillance and early intervention are crucial. Implementing routine screening for MDROs in high-risk TBI patients particularly those who with low GCS scores or comorbidities can facilitate early detection of colonization or infection. Antibiotic stewardship programs (ASPs) are also essential to curbing the development of antimicrobial resistance. Restricting broad-spectrum antibiotics to cases with confirmed susceptibility, guided by local resistance data, and adhering strictly to guideline-recommended duration and dosing can mitigate antibiotic selection pressure and delay the emergence of resistant strains. The resistance patterns observed in this study further emphasize the urgency of implementing ASPs. Infection control measures must be rigorously enforced, including hand hygiene, contact precautions, and environmental disinfection, particularly in intensive care units and surgical wards for TBI patients. Optimizing the management of comorbidities, such as glycemic control in patients with diabetes, may improve immune function and reduce susceptibility to infections. Moreover, efforts to shorten hospital stays can minimize exposure to nosocomial pathogens, which is especially important for infection-prone TBI patients. Finally, regular training for healthcare workers on infection prevention, MDRO management, and antibiotic stewardship, coupled with structured patient and family education on hygiene practices, can help establish a comprehensive framework for infection control.

In summary, this study successfully identified the primary pathogenic bacteria and their resistance characteristics in TBI-related infections, underscored the significance of infection risk factors, and established a basis for developing hospital infection control strategies. Our study still have a limitation, although the sample size of this study was adequate, the CIs for some variables (e.g., prescription of antibiotic) were wide, which may be related to multicollinearity or uneven distribution of variables. In consideration of severe collinearity was ruled out by means of VIF test and VIF values ranged from 1.013 to 2.955 in our study, all well below the commonly used threshold of 5, we support future studies could further refine the type or dose of antibiotics used to improve the precision of estimates.

## Data Availability

The raw data supporting the conclusions of this article will be made available by the authors, without undue reservation.
